# Semiautomated breast ultrasound report generation using multimodal large language models and deep learning

**DOI:** 10.3389/fmed.2026.1679203

**Published:** 2026-01-21

**Authors:** Khadija Azhar, Byoung-Dai Lee, Shi Sub Byon, SeungJae Lee, Kyu Ran Cho, Sung Eun Song

**Affiliations:** 1 AI Laboratory, HealthHub Co., Ltd., Seoul, Republic of Korea; 2 Division of AI and Computer Engineering, Kyonggi University, Suwon, Kyonggi-do, Republic of Korea; 3 Dnotitia Inc., Seoul, Republic of Korea; 4 Department of Radiology, Korea University Anam Hospital, Korea University College of Medicine, Seoul, Republic of Korea

**Keywords:** breast ultrasound, deep learning, elastography, large language model, semiautomated report generation

## Abstract

**Introduction:**

Breast ultrasound (US) imaging is essential for early breast cancer detection, yet generating diagnostic reports is labor-intensive, particularly when incorporating multimodal elastography.

**Methods:**

This study presents a novel framework that combines multimodal large language models and deep learning to generate semiautomated breast US reports. This framework bridges the gap between manual and fully automated workflows by integrating radiologist annotations with advanced image classification and structured report compilation. A total of 2,119 elastography images and 60 annotated patient cases were retrospectively collected from two US machines.

**Results:**

The system demonstrated robust performance in elastography classification, achieving areas under the receiver operating characteristic curve of 0.92, 0.91, and 0.88 for shear-wave, strain, and Doppler images, respectively. In the evaluated dataset, the report generation module correctly identified all suspicious masses across both US machines, achieving 100% sensitivity in lesion detection, with an average report generation time of 31 s per patient using the GE Healthcare machine and 36 s using the Supersonic Image machine.

**Discussion:**

The proposed framework enables accurate, efficient, and device-adaptable breast US report generation by combining multimodal DL and prompt-based LLM inference. It significantly reduces radiologist workload and demonstrates potential for scalable deployment in real-world clinical workflows.

## 1 Introduction

Breast cancer is the most prevalent cancer among women and a leading cause of cancer-related mortality worldwide (1). Early and accurate detection is crucial for improving survival rates, and imaging plays a central role in both diagnosis and management (2). Among various imaging modalities, ultrasound (US) is widely utilized because of its noninvasive nature, affordability, and high sensitivity. B-mode US serves as the cornerstone of breast cancer screening, providing detailed visualization of tissue structures. Recently, US elastography—including shear-wave, strain, and Doppler elastography—has emerged as a valuable complement to B-mode imaging by quantifying tissue stiffness, thereby improving diagnostic accuracy in detecting malignancies (3).

Routine breast US screening requires radiologists to analyze images of the breast and axilla, identify abnormalities, such as cysts or tumors, assess their characteristics (such as size, shape, echotexture, and tissue stiffness), and compile detailed diagnostic reports. This process is time-intensive and requires substantial expertise, as radiologists must manually interpret multimodal imaging data and document findings in a structured format. For instance, generating a single diagnostic report typically takes 5–10 min (4, 5). The inclusion of elastography findings further increases this workload, posing additional challenges for clinical workflows and potentially resulting in delays, inconsistent reporting, and reduced diagnostic quality.

Recent advances in ultrasound automation have been driven largely by Automated Breast Ultrasound (ABUS) systems, which provide standardized, operator-independent whole-breast imaging with three-dimensional reconstruction capabilities (6). ABUS has demonstrated improved cancer detection sensitivity, particularly in women with dense breasts, and minimizes examiner-dependent variability by automating image acquisition (5). These systems also enable consistent image quality, shorten examination time, and allow radiologists to perform retrospective interpretation, thereby improving workflow efficiency (7). In parallel, the integration of artificial intelligence (AI) into ABUS platforms and handheld ultrasound devices has further expanded automation through automated lesion detection, lesion classification, and structured decision-support tools (8). These AI-augmented technologies are increasingly incorporated into clinical practice, demonstrating real-world utility in reducing radiologist workload and accelerating report generation (9). Collectively, these developments reflect a broader transition toward automated, AI-enhanced breast ultrasound workflows designed to improve diagnostic consistency and early detection (10).

Multimodal large language models (LLMs) have further advanced this landscape by enabling structured report generation that integrates imaging data with textual annotations, thereby supporting automated and device-adaptive reporting across heterogeneous ultrasound systems. LLMs have shown strong performance in generating radiology reports for modalities such as MRI, CT, and radiography, highlighting their potential applicability to breast US imaging (11). When combined with automated image acquisition and DL-based image classification, multimodal LLMs form a promising foundation for unified breast US workflows that integrate automation, multimodal interpretation, and streamlined reporting.

Despite these advances, the application of LLM-based reporting and multimodal automation to breast US remains limited. Handheld breast US continues to be highly operator-dependent, and the substantial heterogeneity among devices poses challenges for cross-platform generalizability. Additionally, existing automated systems do not address the full pipeline required for comprehensive reporting, particularly the integration of multiple elastography modalities into unified, structured narrative outputs. Consequently, radiologists must still manually extract tumor size and location annotations, interpret multimodal findings, and assemble detailed reports—resulting in variability, cognitive burden, and workflow inefficiency. This study proposes a novel framework to address these challenges by integrating the multimodal capabilities of LLMs with DL algorithms for semiautomated breast US report generation. The framework incorporates radiologist annotations recorded during routine screenings to enhance accuracy and minimize the need for extensive retraining. Device-specific prompts within the LLM ensure compatibility with diverse breast US machines, eliminating labor-intensive software customization. Additionally, the framework supports automated analysis of multiple elastography modalities, including shear-wave, strain, and Doppler elastography, to provide comprehensive diagnostic information. By bridging the gap between manual and fully automated systems, this approach reduces radiologists’ workloads, streamlines diagnostic workflows, and improves reporting consistency while maintaining high accuracy and reliability in clinical practice.

The contributions of this study are as follows:

- We leveraged multimodal integration to extract and analyze radiologist annotations using LLMs, facilitating seamless adaptation to various breast US machines without requiring extensive retraining.
- We enhanced the semiautomated report generation process by integrating DL algorithms to classify tissue stiffness across multiple elastography modalities, ensuring comprehensive and accurate diagnostic insights.
- We conducted extensive experiments to validate the effectiveness of the framework, demonstrating accurate classification of tissue stiffness and significant reductions in report generation time, thereby confirming its clinical utility.

By advancing the capabilities of LLMs and DL in breast US imaging, this study offers a scalable and practical solution to meet the growing demand for efficient and accurate diagnostic workflows, paving the way for broader AI integration in clinical practice.

## 2 Materials and methods

This retrospective study was approved by the Institutional Review Board of Korea University Anam Hospital, where the research was conducted (Institutional Review Board No. 2024AN0270). All methods adhered to the ethical standards outlined in the Declaration of Helsinki. The need for informed consent was waived by the institutional review board because the data used in this retrospective study had already been fully deidentified to protect patient confidentiality.

### 2.1 Study population and datasets

This study used four distinct datasets to develop and evaluate the proposed framework. Three datasets were used for training and validating the DL models to enable the automatic classification of elastography image types, specifically shear-wave, strain, and Doppler images. These datasets comprised 543 breast US shear-wave images, 816 breast US strain images, and 760 breast US Doppler images. All images were acquired from non-overlapping patients at Korea University Anam Hospital through the Picture Archiving and Communication System between January 2023 and December 2023, using either LOGIQ E10 with a linear-type 18.5 MHz EUP-L75 probe (GE HealthCare, Chicago, IL, United States) or Aixplorer with a linear-type 4–15 MHz transducer (SuperSonic Imagine, Aix-en-Provence, France). Each image was stored in the Digital Imaging and Communication in Medicine (DICOM) format and meticulously annotated by a senior board-certified radiologist specializing in breast tumors.

For model training and inference, the DICOM images were converted into color images in PNG format while retaining their original resolution of approximately 1,292 × 970 pixels. All images were anonymized prior to use to protect patient privacy. The datasets were partitioned into training, validation, and testing subsets based on predefined ratios specific to each dataset, ensuring balanced representation across all classes and preventing data leakage. Details of the dataset distributions are presented in Table 1.

**TABLE 1:** Dataset distribution details for deep learning model development.

Elastography	Class	No. of samples	Training set	Validation set	Test set
Shear-wave	Negative	81	48	17	16
	Equivocal	198	118	41	39
	Positive	264	157	55	52
	Total	543	323 (59%)	113 (21%)	107 (20%)
Strain	Negative	211	127	42	42
	Equivocal	323	195	65	64
	Positive	282	169	56	56
	Total	816	491 (60%)	163 (20%)	162 (20%)
Doppler	Type 1	337	216	53	68
	Type 2	174	112	27	35
	Type 3	249	160	39	50
	Total	760	488 (64%)	119 (16%)	153 (20%)

An additional dataset was constructed to evaluate the performance of the proposed semiautomated breast US report generation system. This dataset, comprising breast US DICOM images paired with the corresponding data annotations and final radiology reports, included scans from 60 patients, evenly distributed between two breast US machines (LOGIQ E10 and Aixplorer). The detailed characteristics of the evaluation datasets are summarized in Table 2. The scans were randomly selected from the Picture Archiving and Communication System at Korea University Anam Hospital, and no patients overlapped with those in the training and validation datasets. Breast US scanning protocols, including equipment and imaging conditions, were consistent with those used for the training and validation datasets. However, the total number of DICOM files and imaging characteristics varied according to lesion size, location, and number. For instance, one patient’s dataset included 58 DICOM images with multiple elastography types (including Doppler elastography, shear-wave elastography, and strain elastography), whereas another patient’s dataset contained 35 DICOM images limited to B-mode and shear-wave imaging.

**TABLE 2:** Summary of evaluation dataset characteristics.

Characteristics	LOGIQ E10	Aixplorer
Number of patients	30	30
Mean total number of US scan images	31.13 (7, 64)	37.13 (5, 70)
Mean number of masses in final radiology reports	5.43 (1, 15)	6.33 (1,13)
Supported US scan image types	B-mode, Shear-wave, Strain, and Doppler images	B-mode, Shear-wave, and Doppler images

Values in parentheses indicate the minimum and maximum of the corresponding metric. US, ultrasound.

### 2.2 System architecture

**FIGURE 1:**
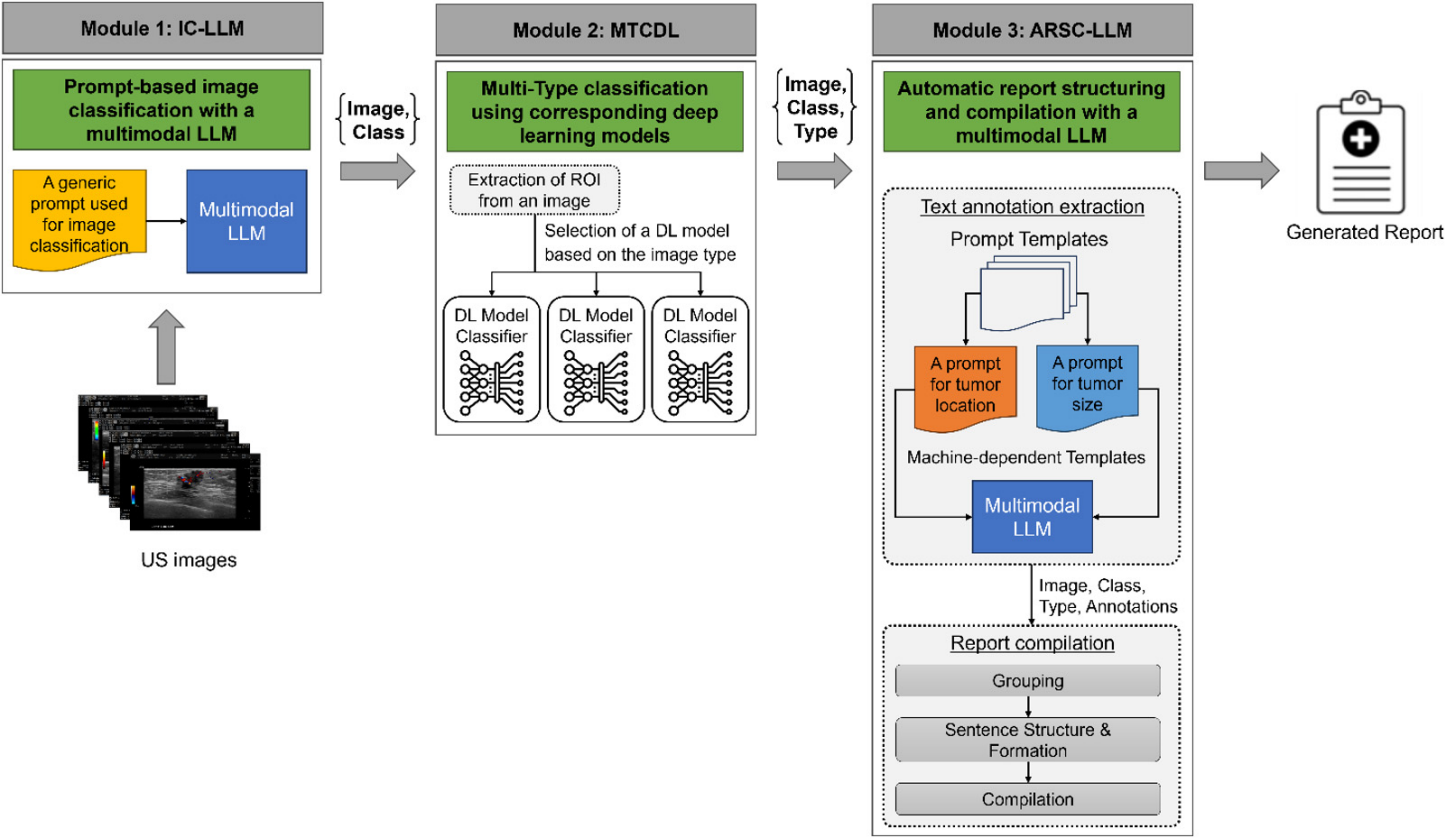
Overview of the proposed semiautomatic breast ultrasound report generation framework, comprising three modules: Image Classification using LLM (IC-LLM), Multi-Type Classification via Deep Learning (MTCDL), and Automatic Report Structuring and Compilation using LLM (ARSC-LLM).

As illustrated in Figure 1, the proposed pipeline comprises three key modules, each serving distinct functions: Image Classification using LLM (IC-LLM), Multi-Type Classification via DL (MTCDL), and Automatic Report Structuring and Compilation using LLM (ARSC-LLM).

#### 2.2.1 IC-LLM module

**FIGURE 2:**
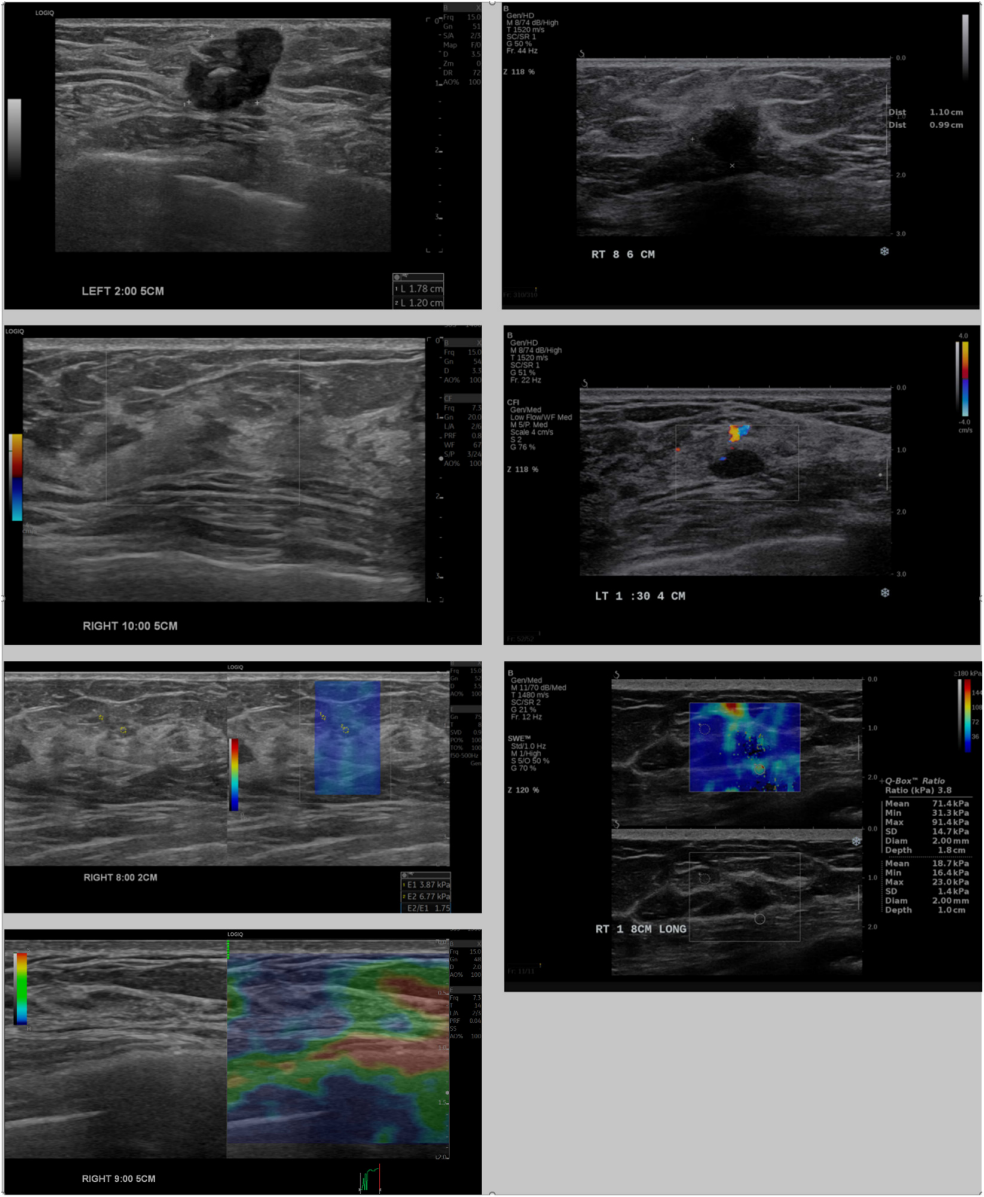
Comparison of breast ultrasound images acquired from LOGIQ E10 (left) and Aixplorer (right). Rows show, from top to bottom: B-mode images with annotated mass sizes, Doppler images, shear-wave elastography, and strain elastography (available only for LOGIQ E10).

The IC-LLM module classifies breast US scans into four imaging types: B-mode, shear-wave elastography, strain elastography, and Doppler elastography, each characterized by distinct visual attributes. For instance, B-mode images lack color legends, whereas shear-wave elastography images include the text “kPa.” Doppler images feature irregular contours with units, such as cm/s, whereas strain elastography images display colored bars with units, such as s/h. Figure 2 illustrates these visual distinctions, highlighting variations in annotation placement, location, and depth information across different US machines.

Unlike conventional image-processing algorithms that rely on predefined rules or manually engineered heuristics, the IC-LLM module performs prompt-based image analysis using the LLM. Leveraging its natural language understanding and visual reasoning capabilities, the LLM systematically identifies semantic cues that distinguish the four scan types. A tailored prompt guides the model to detect features such as measurement scales, color legends, textual labels, color patterns, and unit markers. Importantly, the LLM does not perform pixel-level feature extraction; rather, it focuses on high-level, visually interpretable semantic cues that are robust across devices. This design enables accurate and scalable classification while remaining independent of specific ultrasound manufacturers.

**FIGURE 3:**
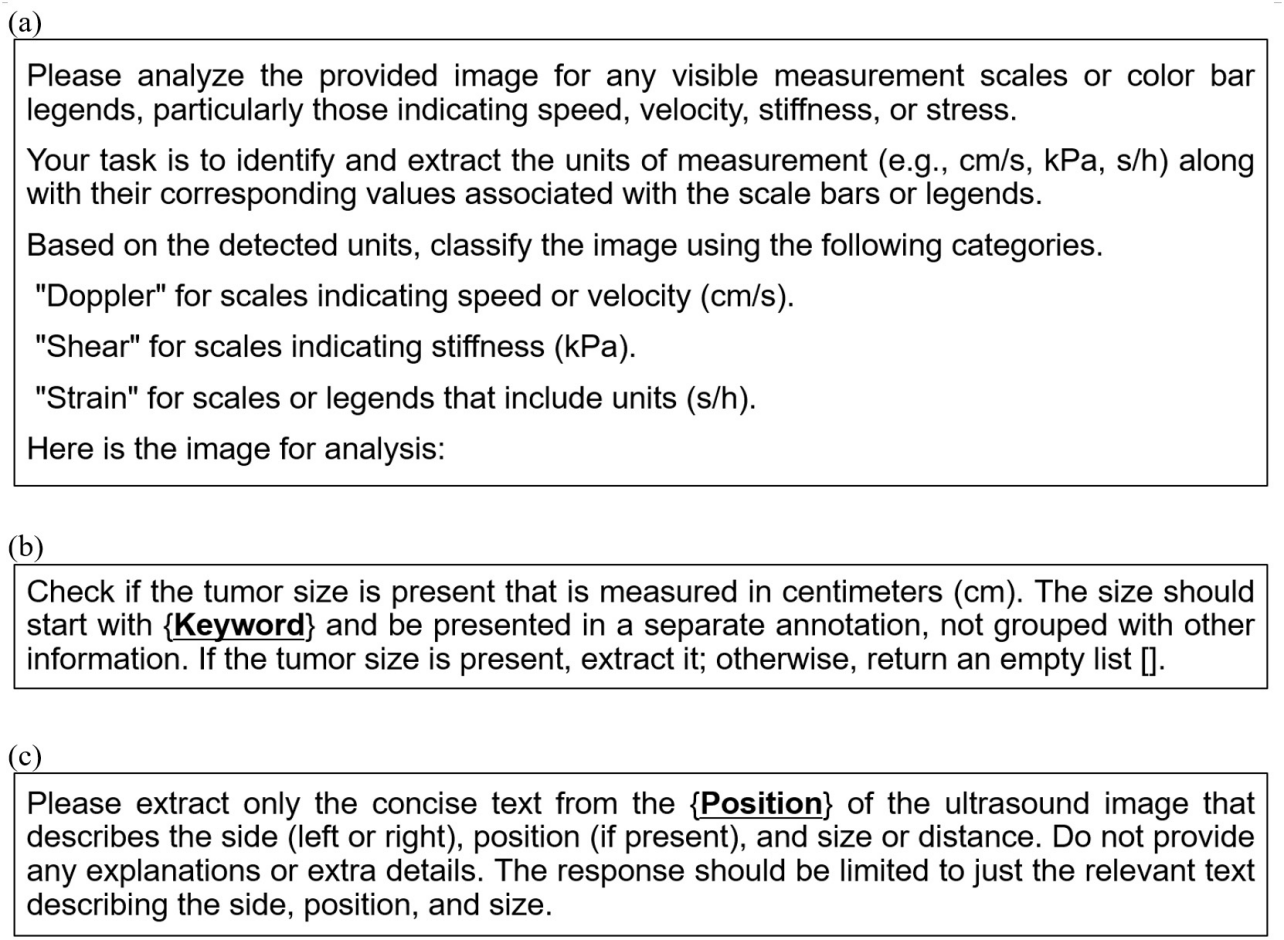
Prompt templates used in the proposed system: **(a)** A generic prompt for the IC-LLM module, applicable across different ultrasound machines, **(b)** a prompt template with curly-brace placeholders, used in the ARSC-LLM module for extracting tumor size, and **(c)** a prompt template with curly-brace placeholders for extracting tumor location in the ARSC-LLM module. These placeholders (e.g., {*Keyword*}, {*Position*}) are automatically populated with machine-specific values retrieved from configuration files to ensure consistent text extraction across different ultrasound devices.

In this study, we utilized GPT-4o min (12) as the multimodal LLM with a prompt for image classification, as shown in Figure 3a. This prompt was designed to be independent of the breast US machines used (i.e., LOGIQ E10 and Aixplorer) to ensure compatibility across different systems. Classification results were annotated directly onto the images and passed to downstream modules, including the MTCDL and ARSC-LLM modules.

The LLM-based approach has several advantages. By leveraging the model’s contextual understanding, it achieves high classification accuracy. Its scalability allows seamless adaptation to new imaging categories through simple modifications to the input prompt. Furthermore, it enhances efficiency by eliminating the need for complex rule-based algorithms, thereby reducing computational overhead.

#### 2.2.2 MTCDL module

The MTCDL module comprises three submodules, each designed to classify specific elastography image modalities. Although the operational framework remained consistent across all image types, the algorithms were tailored to accommodate the unique characteristics of each modality to ensure accurate and reliable classification. Upon receiving elastography images and their associated scan types from the IC-LLM module, the MTCDL module extracts regions of interests specific to the identified scan type. These regions of interests were then processed using DL models optimized for each elastography modality, including shear-wave, strain, and Doppler elastography. This modular and adaptable structure allows MTCDL to handle diverse elastography image types effectively, ensuring precise analysis of key diagnostic features. By seamlessly integrating this module into the report-generation workflow, the MTCDL module reduces the workload of radiologists and enhances the efficiency of automated breast US workflows.

This study does not aim to develop new architectures; rather, it focuses on validating the feasibility and effectiveness of the proposed framework as a proof of concept. To this end, we leveraged well-established DL models, including ResNet (13), GoogLeNet (14), DenseNet (15), and EfficientNet (16). These models were selected because of their reliability and proven performance in classification tasks, thus providing acceptable accuracy for this application. By demonstrating its effectiveness in elastographic image classification, the proposed framework highlights its potential to streamline the report generation process and significantly reduce the time and effort required for manual interpretation.

#### 2.2.3 ARSC-LLM module

The ARSC-LLM module is the final component of the proposed system and is designed to perform two sequential functions. First, it extracts text annotations from US images, including tumor sizes annotated by radiologists during routine screenings, and automatically records details, such as the three-dimensional location of lesions. Second, these annotations are integrated with the elastography images and their corresponding types provided by the MTCDL module to generate a comprehensive final report.

During the text annotation extraction phase, the module employs prompt-based image analysis using a multimodal LLM, similar to the IC-LLM module. As illustrated in Figure 2, the heterogeneous characteristics of US scan image types and machines make it challenging to create a single generic prompt. To address this issue, our approach dynamically generates machine-specific prompts using placeholder-based prompt templates. These templates, designed to extract the tumor size and three-dimensional location, contain placeholders for machine-specific details that are automatically populated using configuration files. The tailored prompts are then used as inputs to the multimodal LLM, enabling the accurate extraction of relevant textual information from US images. This method ensures robust and adaptable text extraction across diverse breast US machines. Figures 3b,c illustrate the prompt templates used to extract location and size annotations.

The report compilation phase organizes US scans into groups based on location details, such as side (including LEFT or RIGHT), clock-face position, and depth, consolidating scans with identical location attributes into cohesive groups. For instance, scans labeled “LEFT 2:00 5 cm” and “LEFT 2:00 5 cm LO” are treated as a single group to streamline analysis. To ensure consistency and clinical relevance, this study considered only the transverse and longitudinal scans for grouping. Although other orientations were not included in the current framework, they can be readily integrated into future research.

To summarize the key findings for each group, the ARSC-LLM module utilizes a predefined sentence template: *“[size] mass in [location] from nipple, Doppler [class], Shear-wave Elastography [class], Strain Elastography [class], Category [BI-RADS category]*.” The system automatically populates size, location details, scan type, and classification information, whereas the Breast Imaging Reporting and Data System (BI-RADS) category, representing categories one to six of BI-RADS, is manually entered by a radiologist based on their interpretation of the images. Using this predefined template, the ARSC-LLM module generates descriptive sentences for each scan group by integrating the cleaned location and size data with the corresponding elastography classifications. For instance, a sample output might read: “*0.41 × 1.2 cm mass in RIGHT 9 h, 6 cm from nipple, Doppler (negative), Shear-wave Elastography (positive), Strain Elastography (equivocal), Category ()*.” In this example, the blank parentheses indicate fields requiring manual input from a radiologist, such as the BI-RADS category. Once generated, sentences are compiled into cohesive text files to form the final report. This approach ensures systematic, accurate, and consistent reporting, while significantly reducing the radiologist’s workload and streamlining the workflow.

### 2.3 Training details

To train the DL models for automatic classification of elastography image types, we utilized TensorFlow 2.6.0, DenseNet-121, GoogleNet, ResNet-50, and EfficientNet-B6 architectures. All models were initialized with pretrained weights and optimized using the Adam optimizer (17) with an initial learning rate of 0.001. A learning rate scheduler and early stopping were employed to refine the training process and enhance model convergence. Each model was trained for 50 epochs with a batch size of eight, progressively reducing the learning rate to ensure performance stability.

The input images were resized to 224 × 224 pixels while preserving aspect ratios. For DenseNet-121, the last five layers were unfrozen for fine-tuning, and the learning rate was adjusted to 0.0001. ResNet-50, GoogleNet, and EfficientNet-B6 followed a similar training procedure, maintaining an initial learning rate of 0.001 to achieve consistent optimization across the architectures.

Although various affine transformation-based data augmentation techniques were tested during training (such as rotation, scaling, and flipping), we observed minimal performance differences between models trained with and without data augmentation. Therefore, the results in this study were based on models trained without data augmentation, ensuring a simpler and more interpretable evaluation process.

The training process was conducted on a system running Ubuntu 22.04.4 LTS, equipped with an Intel Core i5 (8th Gen) CPU and an NVIDIA RTX 2060 GPU. The software environment included Python 3.5, CUDA 10, CuDNN 7.6.5, TensorFlow 2.6.0, and Keras 2.1.0, providing a robust computational setup for model development and evaluation.

## 3 Results

### 3.1 Performance of the DL models

The diagnostic performance of the DL models was evaluated using multiple metrics, including accuracy, precision, recall, F1-score, and the area under the receiver operating characteristic curve (AUROC). These metrics were selected to comprehensively assess the ability of each model to identify and classify elastography image types and to provide insights into both their discriminative capability and clinical utility. In addition, pairwise DeLong tests (18) were conducted to evaluate the statistical significance of differences in AUROC among the models.

Table 3 summarizes the overall performance of ResNet-50, GoogleNet, DenseNet-121, and EfficientNet-B6 across three US scan types: shear-wave, strain, and Doppler elastography. DenseNet-121 exhibited the best classification performance for shear-wave images, achieving the highest values across all evaluation metrics. For strain and Doppler images, GoogleNet consistently outperformed the other models, delivering the best results across all metrics.

**TABLE 3:** Performance of deep learning models in elastography image classification.

Elastography	Models	Accuracy	Precision	Recall	F1-score
Shear-wave	ResNet-50	0.41	0.57	0.32	0.35
	GoogLeNet	0.81	0.79	0.80	0.80
	DenseNet-121	0.87	0.87	0.85	0.86
	EfficientNet-B6	0.71	0.76	0.65	0.67
Strain	ResNet-50	0.52	0.52	0.49	0.62
	GoogLeNet	0.79	0.79	0.79	0.79
	DenseNet-121	0.52	0.50	0.48	0.65
	EfficientNet-B6	0.51	0.49	0.49	0.58
Doppler	ResNet-50	0.58	0.56	0.51	0.50
	GoogLeNet	0.76	0.76	0.77	0.75
	DenseNet-121	0.64	0.62	0.63	0.59
	EfficientNet-B6	0.56	0.49	0.56	0.50

**FIGURE 4:**
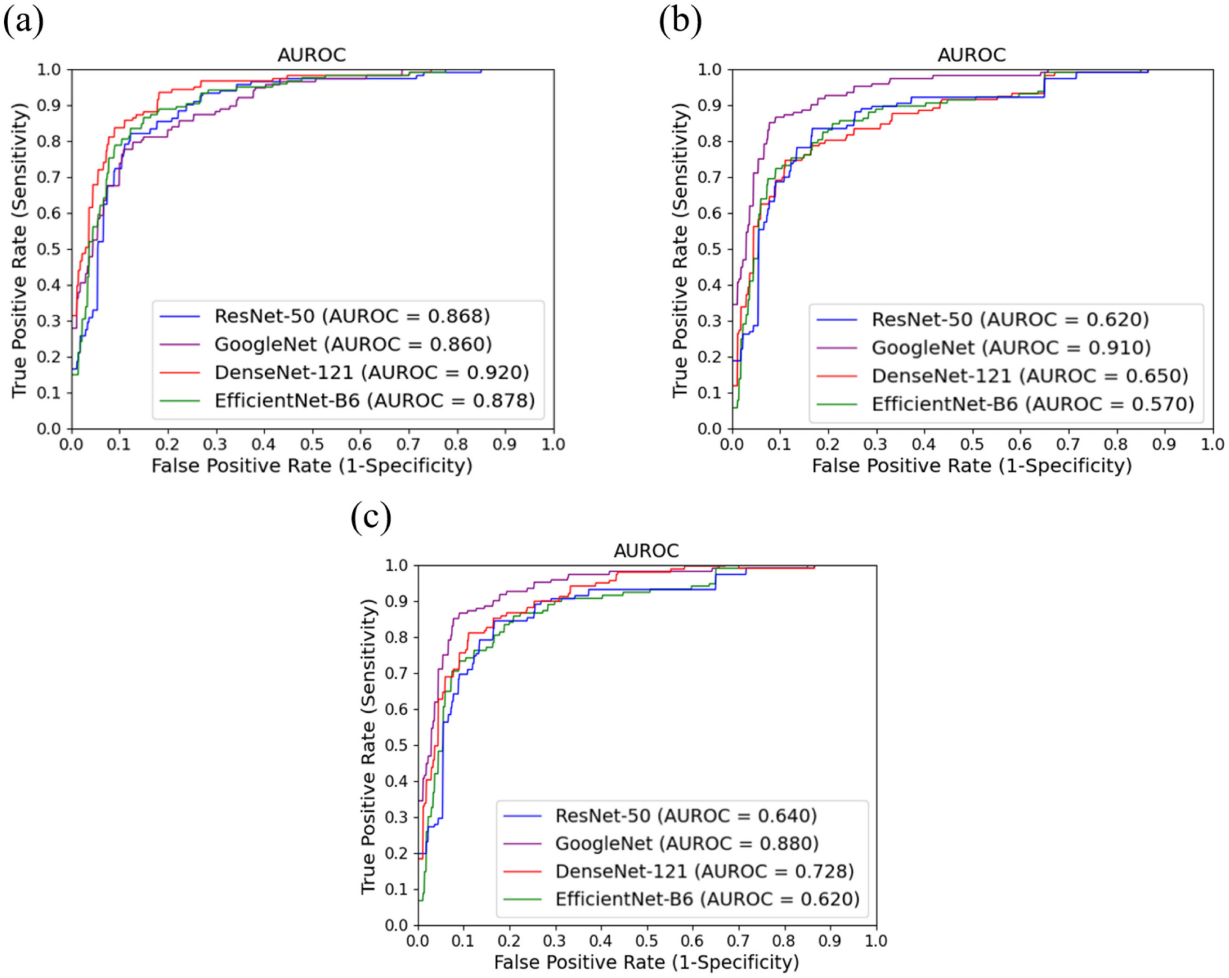
AUROC curves for three ultrasound modalities: **(a)** Shear-wave elastography images, **(b)** strain elastography images, and **(c)** Doppler imaging.

**FIGURE 5:**
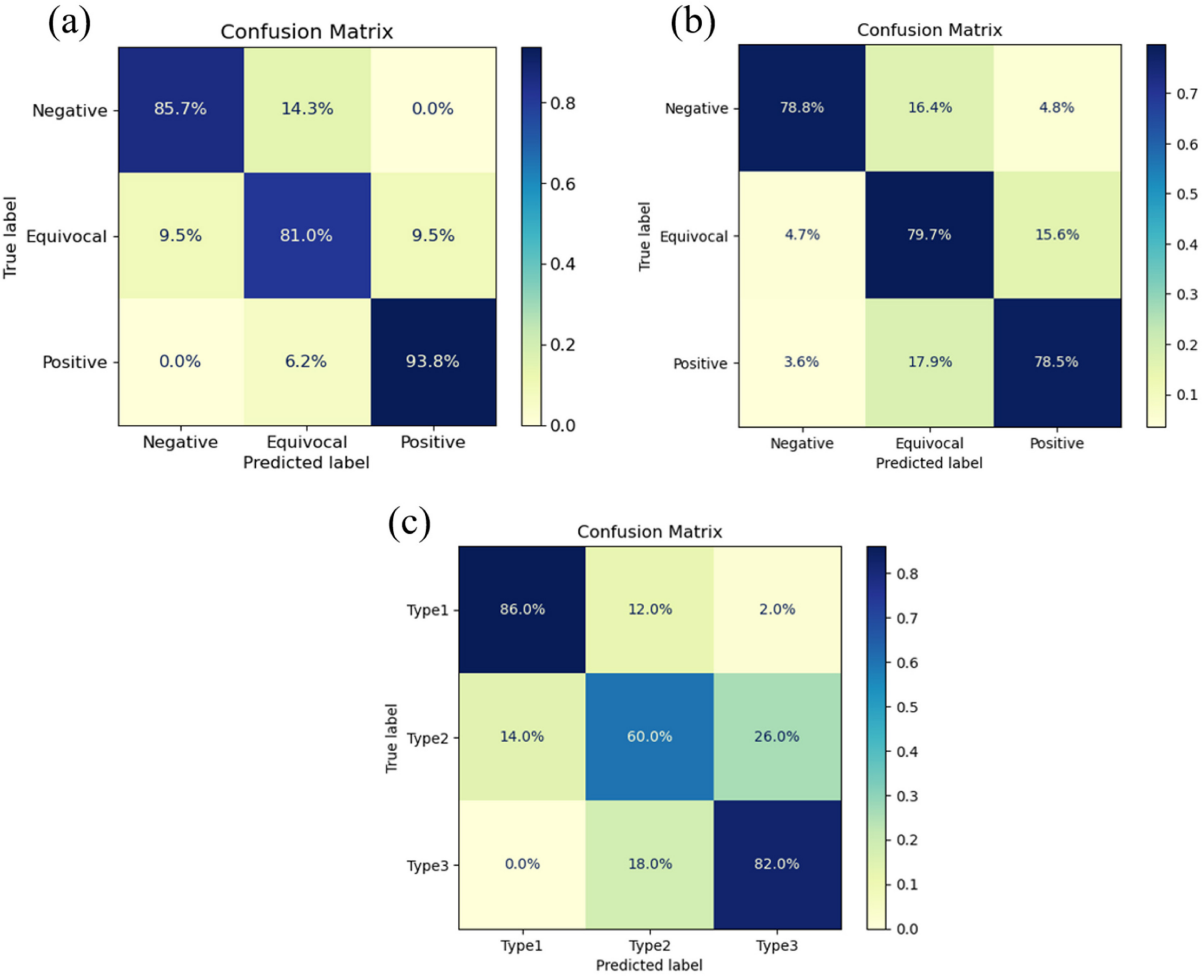
Confusion matrices for the best-performing deep learning models across three ultrasound modalities: **(a)** DenseNet-121 for shear-wave elastography, **(b)** GoogleNet for strain elastography, and **(c)** GoogleNet for Doppler imaging.

These findings were further validated by the AUROC curves presented in Figure 4, which complement the performance metrics. DenseNet-121 achieved an AUROC of 0.92 [95% confidence interval (CI): 0.87–0.97] for shear-wave images, whereas GoogleNet demonstrated superior performance for strain and Doppler images, with AUROCs of 0.91 (95% CI: 0.85–0.95) and 0.88 (95% CI: 0.81–0.93), respectively. Consistent with these AUROC trends, the DeLong test summarized in Table 4 showed that DenseNet-121 significantly outperformed GoogleNet, ResNet-50, and EfficientNet-B6 in the shear-wave modality (*p* = 0.008, *p* = 0.045, and *p* = 0.030, respectively). For both strain and Doppler elastography, GoogleNet exhibited statistically significant superiority over all other models—DenseNet-121, ResNet-50, and EfficientNet-B6—with all corresponding pairwise comparisons yielding *p* < 0.001. Additionally, Figure 5 presents the confusion matrices for the best-performing models for each elastography image type, providing detailed insights into classification accuracy and error distributions.

**TABLE 4:** Statistical significance of model performance differences across elastography modalities based on the DeLong Test.

Elastography	Model 1	Model 2	*P*-value
Shear-wave	DenseNet-121	GoogleNet	0.008
	DenseNet-121	ResNet-50	0.045
	DenseNet-121	EfficientNet-B6	0.030
	GoogleNet	ResNet-50	0.650
	GoogleNet	EfficientNet-B6	0.420
	ResNet-50	EfficientNet-B6	0.580
Strain	GoogleNet	DenseNet-121	< 0.001
	GoogleNet	ResNet-50	< 0.001
	GoogleNet	EfficientNet-B6	< 0.001
	DenseNet-121	ResNet-50	0.320
	DenseNet-121	EfficientNet-B6	0.150
	ResNet-50	EfficientNet-B6	0.220
Doppler	GoogleNet	DenseNet-121	0.010
	GoogleNet	ResNet-50	< 0.001
	GoogleNet	EfficientNet-B6	< 0.001
	DenseNet-121	ResNet-50	0.120
	DenseNet-121	EfficientNet-B6	0.050
	ResNet-50	EfficientNet-B6	0.480

### 3.2 Evaluation of automatic report generation

As described in the previous section, breast US scan images of patients with identical location and depth attributes were grouped to streamline the report generation process. Each group included multiple elastography types (including shear-wave, strain, or Doppler), depending on the imaging data. The ARSC-LLM module integrates these annotations to generate reports for groups containing radiologist annotations recorded during routine screening. This grouping ensures consistency in report generation by associating all relevant scan data and annotations within a unified structure.

To evaluate the system, the accuracy of the generated reports was compared with that of the ground truth (GT) reports prepared by radiologists. The evaluation focused on suspicious masses documented in the GT reports, with key accuracy criteria including location, depth, and elastography type associated with each mass. A report was deemed accurate if all attributes matched the corresponding GT details, ensuring a rigorous assessment of system performance.

Table 5 summarizes the performance of the proposed system across 60 patient scans from two different US machines (LOGIQE10 and Aixplorer), ensuring a diverse and representative dataset. The total number of scanned images corresponded to the complete count of US images recorded for each patient. These images were grouped based on shared location and depth attributes, yielding a total number of groups. Among these, groups containing suspicious masses documented in the GT reports were classified as “groups for suspicious masses.” The number of correctly generated reports represented the system-generated reports that accurately aligned with GT entries, serving as a key indicator of system accuracy. The ratio was calculated as the number of correctly generated reports divided by the number of groups for suspicious masses, with a ratio of one indicating perfect system performance. Additionally, the execution time measured the total duration from the input of a patient’s DICOM files to the completion of the final report, reflecting the system’s operational efficiency.

**TABLE 5:** Performance of the proposed system in generating accurate reports across different ultrasound machines.

Performance metrics	LOGIQ E10	Aixplorer
Mean total number of US scan images	31.13 (7.0, 64.0)	37.13 (5.0, 70.0)
Mean total number of groups	7.30 (2.0, 21.0)	6.34 (2.0, 15.0)
Mean number of groups for suspicious masses	5.10 (1.0, 15.0)	4.67 (1.0, 13.0)
Mean number of correctly generated reports	5.10 (1.0, 15.0)	4.67 (1.0, 13.0)
Mean ratio	1.0 (1.0, 1.0)	1.0 (1.0, 1.0)
Mean execution time (s)	31.31 (11.1, 58.1)	36.30 (10.0, 59.9)

Values in parentheses indicate the minimum and maximum of the corresponding metric. US, ultrasound.

The results in Table 5 highlight the robustness and consistency of the system across both US machines. It achieved a 100% accuracy rate for generating reports on suspicious masses, with a ratio of 1.0 for both the GE and Supersonic machines. Variations in the number of scanned images, groups, and suspicious masses per patient did not affect system accuracy, demonstrating its adaptability to diverse datasets and imaging protocols. This finding underscores the system’s ability to consistently generate accurate and reliable reports, regardless of the imaging machine or scanning conditions.

The execution time for report generation remained efficient, averaging 31 s for LOGIQ E10 and 36 s for Aixplorer, even when using low-performance GPUs. This efficiency highlights the system’s scalability and practicality for integration into diverse clinical workflows, including those with limited computational resources. The detailed performance evaluation results for each patient are presented in Supplementary Tables 1, 2.

**FIGURE 6:**
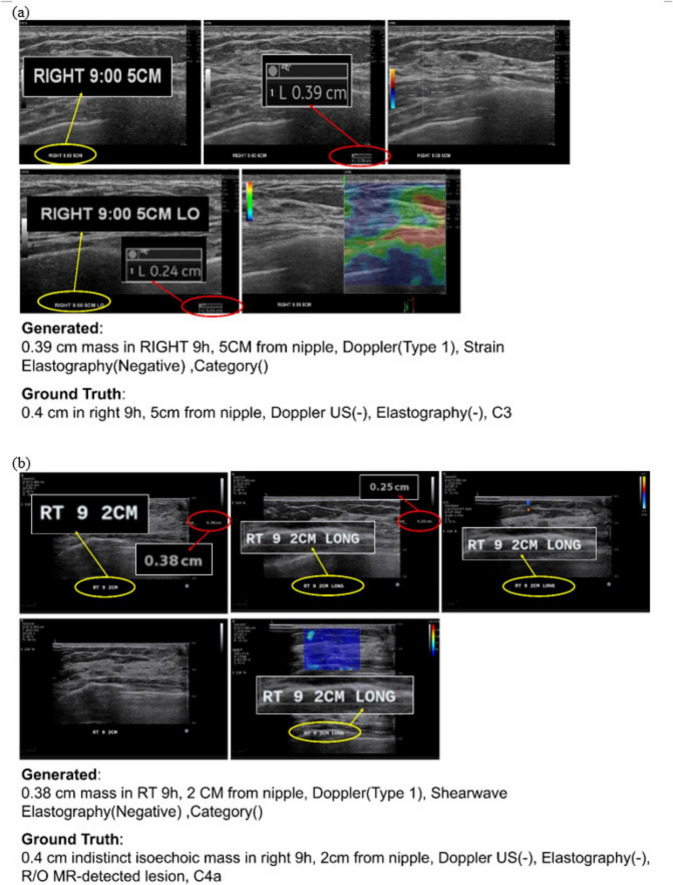
Examples of automatically generated reports for suspicious breast masses. Shown are ultrasound images acquired with **(a)** LOGIQ E10 and **(b)** Aixplorer, together with the corresponding ground truth and the report generated by the proposed system.

Figure 6 presents examples of automatically generated reports based on DICOM files from patients scanned using both US machines. The figure illustrates the system’s ability to classify imaging modalities, extract annotations, and compile structured reports without requiring machine-specific customization. This scalability and adaptability across imaging systems validate the potential of the proposed framework to standardize and streamline diagnostic workflows in diverse clinical environments.

## 4 Discussion and conclusion

This study presented a novel framework that leverages LLMs and DL to enable semiautomatic breast US report generation. This framework bridges the gap between manual and fully automated workflows by integrating radiologist annotations with advanced image classifications and structured report compilations. A key feature of this system is its adaptability to heterogeneous breast US machines, achieved through machine-specific prompts within the LLM. This prompt-based strategy externalizes device adaptation from model parameters to structured semantic guidance, enabling zero-shot generalization across vendors without retraining and providing a scalable alternative to rule-based or fine-tuning–dependent pipelines. By avoiding labor-intensive software customization, the framework ensures compatibility across diverse clinical environments while maintaining high reporting quality. The semiautomated process not only reduces radiologists’ workloads but also enhances reporting consistency.

As part of the report-generation workflow, the framework incorporates DL models to automate elastography image classification. DenseNet-121 demonstrated the best performance for shear-wave images, achieving an AUROC of 0.92, whereas GoogleNet outperformed other models for strain and Doppler elastography, with AUROCs of 0.91 and 0.88, respectively. Despite the relatively small dataset size and inherent class imbalance, the system consistently exhibited robust performance across all elastography modalities. Additionally, the framework achieved 100% accuracy in generating reports on suspicious masses across two different US machines, with a ratio of 1.0 for all cases. Report generation was highly efficient, with an average execution time of approximately 31 s per patient for LOGIQ E10 and 36 s per patient for Aixplorer, even when using low-performance GPUs. These results validate the reliability and efficiency of the proposed framework and underscore its potential to streamline clinical workflows, enhance efficiency, and improve the diagnostic accuracy of breast US imaging.

Recent advancements in AI have spurred various approaches to automated report generation, ranging from feature extraction to comprehensive text generation using pretrained LLMs. For example, Yang et al. (4) demonstrated an improvement over state-of-the-art image-captioning methods by leveraging convolutional neural networks and attention mechanisms for feature extraction, followed by Long Short-Term Memory for report generation. However, their reliance on the highest-quality images limits generalizability. Similarly, Huh et al. (19) proposed a system using LangChain (20) and LLMs for breast US reporting, which showed promising results in clinical evaluations but relied on rule-based image splitting, potentially restricting adaptability to diverse imaging conditions. Ge et al. (5) integrated structured categories and AI classifiers for personalized reporting but heavily depended on manual refinement for malignant cases. By contrast, our framework automates both image classification and structured report generation, reducing the need for extensive physician refinement. Furthermore, its use of machine-specific prompts ensures seamless compatibility across diverse imaging systems, maintaining high accuracy and consistency without requiring labor-intensive customization.

Despite its strengths, this study had several limitations. First, the dataset was limited to images from two breast US machines (LOGIQ E10 and Aixplorer), which may not have captured the full range of variations in imaging equipment. Expanding the dataset to include additional machines and vendors would enhance the system’s generalizability. Second, the framework relies on radiologist-provided annotations (e.g., lesion size and location) embedded in DICOM images for report generation. While this ensures clinical relevance and avoids the need for pixel-level segmentation, it also introduces annotation dependence—meaning the system cannot operate autonomously when such inputs are incomplete, inconsistent, or absent. Future work should integrate automated lesion detection and segmentation models to extract these attributes directly from raw images, enabling a fully end-to-end reporting pipeline. Third, while the prompt-based LLM design facilitates adaptation across different ultrasound devices, its performance remains sensitive to prompt structure, imaging layout, and vendor-specific labeling conventions. The current placeholder-based templates generalized well across LOGIQ E10 and Aixplorer; however, they may be less robust to non-standard interface variations such as differing font styles, handwritten labels, or non-DICOM overlays. Adaptive prompt learning or few-shot prompt optimization may offer greater resilience to such variations. Fourth, although the DL models demonstrated robust performance, their accuracy could be further improved through larger and more diverse datasets and advanced techniques, such as ensemble learning or fine-tuning on task-specific data. Finally, despite the efficient execution times observed in our experiments, large-scale deployment under high patient volumes warrants further evaluation to ensure scalability and operational feasibility in busy clinical workflows.

In conclusion, this study demonstrated that the proposed framework effectively addresses key challenges in breast US report generation by integrating LLMs and DL models into a semiautomated workflow. The high accuracy, adaptability, and efficient processing time of the system underscore its potential for standardizing and streamlining diagnostic workflows. This framework offers a scalable solution for diverse clinical settings by reducing the workload of radiologists and enhancing reporting consistency. Future research should prioritize expanding compatibility with additional imaging systems, exploring fully automated solutions and validating the framework in largescale clinical environments.

## Data Availability

The original contributions presented in the study are included in the article/Supplementary material, further inquiries can be directed to the corresponding author.
