# Supplementary material for: Semiautomated breast ultrasound report generation using multimodal large language models and deep learning
Source: Front Med (Lausanne). 2026 Jan 21;13:1679203. doi: 10.3389/fmed.2026.1679203 (PMC12867921; doi:10.3389/fmed.2026.1679203)
Supplement: Supplementary file 1 [file Data_Sheet_1.docx]

**Supplementary Information**

**Supplementary Table 1.** Performance Evaluation of the Automatic Report Generation System for Individual Patients Scanned Using the LOGIQ E10.

| **Patient ID** | **Total no. of scan images** | **Total no. of groups** | **No. of groups for suspicious masses** | **No. of correctly generated reports** | **Ratio** | **Execution Time (sec.)** |
| --- | --- | --- | --- | --- | --- | --- |
| Patient-1 | 18 | 3 | 2 | 2 | 1 | 11.01 |
| Patient-2 | 31 | 4 | 2 | 2 | 1 | 35.10 |
| Patient-3 | 35 | 7 | 7 | 7 | 1 | 34.41 |
| Patient-4 | 28 | 4 | 2 | 2 | 1 | 29.12 |
| Patient-5 | 36 | 14 | 13 | 13 | 1 | 33.21 |
| Patient-6 | 41 | 8 | 3 | 3 | 1 | 46.12 |
| Patient-7 | 24 | 3 | 2 | 2 | 1 | 30.12 |
| Patient-8 | 24 | 4 | 1 | 1 | 1 | 30.23 |
| Patient-9 | 49 | 12 | 11 | 11 | 1 | 37.11 |
| Patient-10 | 22 | 4 | 2 | 2 | 1 | 22.10 |
| Patient-11 | 24 | 5 | 2 | 2 | 1 | 27.50 |
| Patient-12 | 44 | 8 | 7 | 7 | 1 | 39.89 |
| Patient-13 | 14 | 2 | 1 | 1 | 1 | 19.12 |
| Patient-14 | 17 | 3 | 2 | 2 | 1 | 20.71 |
| Patient-15 | 35 | 6 | 2 | 2 | 1 | 32.11 |
| Patient-16 | 27 | 5 | 3 | 3 | 1 | 28.30 |
| Patient-17 | 24 | 6 | 3 | 3 | 1 | 26.90 |
| Patient-18 | 30 | 5 | 3 | 3 | 1 | 30.04 |
| Patient-19 | 64 | 21 | 15 | 15 | 1 | 55.01 |
| Patient-20 | 25 | 5 | 4 | 4 | 1 | 28.90 |
| Patient-21 | 46 | 8 | 5 | 5 | 1 | 36.85 |
| Patient-22 | 58 | 13 | 1 | 1 | 1 | 38.30 |
| Patient-23 | 38 | 7 | 5 | 5 | 1 | 36.00 |
| Patient-24 | 32 | 12 | 12 | 12 | 1 | 37.23 |
| Patient-25 | 7 | 2 | 2 | 2 | 1 | 15.30 |
| Patient-26 | 23 | 11 | 11 | 11 | 1 | 33.00 |
| Patient-27 | 56 | 11 | 9 | 9 | 1 | 58.12 |
| Patient-28 | 19 | 8 | 7 | 7 | 1 | 21.00 |
| Patient-29 | 52 | 10 | 8 | 8 | 1 | 49.99 |
| Patient-30 | 51 | 8 | 6 | 6 | 1 | 47.51 |

**Supplementary Table 2.** Performance Evaluation of the Automatic Report Generation System for Individual Patients Scanned Using the Aixplorer.

| **Patient ID** | **Total no. of scan images** | **Total no. of groups** | **No. of groups for suspicious masses** | **No. of correctly generated reports** | **Ratio** | **Execution Time (sec.)** |
| --- | --- | --- | --- | --- | --- | --- |
| Patient-1 | 16 | 3 | 2 | 2 | 1 | 30.01 |
| Patient-2 | 22 | 3 | 3 | 3 | 1 | 38.21 |
| Patient-3 | 23 | 6 | 4 | 4 | 1 | 27.99 |
| Patient-4 | 30 | 5 | 3 | 3 | 1 | 28.39 |
| Patient-5 | 25 | 4 | 2 | 2 | 1 | 34.28 |
| Patient-6 | 53 | 8 | 7 | 7 | 1 | 49.35 |
| Patient-7 | 23 | 4 | 3 | 3 | 1 | 29.12 |
| Patient-8 | 40 | 7 | 5 | 5 | 1 | 30.96 |
| Patient-9 | 34 | 5 | 3 | 3 | 1 | 40.02 |
| Patient-10 | 46 | 8 | 6 | 6 | 1 | 34.07 |
| Patient-11 | 5 | 2 | 1 | 1 | 1 | 10.00 |
| Patient-12 | 70 | 15 | 13 | 13 | 1 | 59.85 |
| Patient-13 | 55 | 8 | 7 | 7 | 1 | 45.01 |
| Patient-14 | 32 | 4 | 2 | 2 | 1 | 40.61 |
| Patient-15 | 25 | 3 | 1 | 1 | 1 | 35.62 |
| Patient-16 | 42 | 7 | 4 | 4 | 1 | 49.11 |
| Patient-17 | 33 | 4 | 2 | 2 | 1 | 27.03 |
| Patient-18 | 20 | 3 | 3 | 3 | 1 | 23.08 |
| Patient-19 | 35 | 9 | 6 | 6 | 1 | 27.23 |
| Patient-20 | 41 | 7 | 6 | 6 | 1 | 45.11 |
| Patient-21 | 34 | 8 | 6 | 6 | 1 | 27.89 |
| Patient-22 | 55 | 9 | 8 | 8 | 1 | 59.01 |
| Patient-23 | 24 | 4 | 2 | 2 | 1 | 32.11 |
| Patient-24 | 51 | 8 | 5 | 5 | 1 | 44.44 |
| Patient-25 | 36 | 7 | 5 | 5 | 1 | 40.98 |
| Patient-26 | 50 | 8 | 5 | 5 | 1 | 37.72 |
| Patient-27 | 36 | 5 | 3 | 3 | 1 | 25.40 |
| Patient-28 | 60 | 13 | 11 | 11 | 1 | 59.12 |
| Patient-29 | 65 | 11 | 9 | 9 | 1 | 59.92 |
| Patient-30 | 33 | 5 | 3 | 3 | 1 | 25.02 |
